# Effect of Electrode Shape and Flow Conditions on the Electrochemical Detection with Band Microelectrodes

**DOI:** 10.3390/s18103196

**Published:** 2018-09-21

**Authors:** Maher Al Khatib, Marco Bellini, Rebecca Pogni, Andrea Giaccherini, Massimo Innocenti, Francesco Vizza, Alessandro Lavacchi

**Affiliations:** 1 Department of Biotechnology, Chemistry and Pharmacy, University of Siena, 53100 Siena, Italy; maher.alkhatib@student.unisi.it (M.A.K.); rebecca.pogni@unisi.it (R.P.); 2 CSGI (Consorzio per lo Sviluppo dei Sistemi a Grande Interfase), 50019 Florence, Italy; 3 Institute for the Chemistry of Organometallic Compounds, Italian National Council for Research, 50019 Florence, Italy; marco.bellini@iccom.cnr.it (M.B.); francesco.vizza@iccom.cnr.it (F.V.); 4 Department of Chemistry, University of Florence, Via della Lastruccia 3-13, 50019 Sesto Fiorentino (Florence), Italy; andrea.giaccherini@unifi.it (A.G.); minnocenti@unifi.it (M.I.)

**Keywords:** electrochemical sensors, microelectrodes, mass transport, diffusion convection

## Abstract

In this work, we report the analysis of the electrochemical detection of electroactive species with band microelectrodes that operate under controlled convection. The study focuses on the determination of the collection efficiency of the analyte as a function of inlet flow velocity and microband geometry (inlaid, bumped and recessed), also providing a straightforward method for the theoretical determination of the lower detection limit. The analysis has been carried out by simulating the dimensionless mass transport with the finite element method, delivering the stationary limiting current density. Simulations have been performed on systems consisting of single and double band electrodes to investigate the trail effect on the electrochemical detection. We show that the obtained dimensionless results can be easily turned into dimensional data, providing a tool for the design of devices. The proposed method is general and can easily be extended to systems with different geometry.

## 1. Introduction

Electrochemical detection with microelectrodes can either complement other analytical techniques (e.g., chromatography) or be used as a stand-alone analytical solution [1,2,3,4,5,6,7,8]. The use of microelectrodes allows high signal to noise ratio and short electrodic current equilibration times compared to macroelectrodes. Additionally, microelectrodes can be easily implemented in electro-analytical instrumentation where the µL scale must be used, as is the case with lab-on-chip devices [9,10,11,12,13,14,15,16,17,18,19,20,21,22,23,24,25,26,27,28].

Flow microelectrodes can be schematically represented as a channel with electroactive species solution flowing in, and with electrodes lying at the bottom; three major geometries are usually encountered: bumped, inlaid, and recessed [29,30,31].

To build efficient devices, it is necessary to consider how physical or geometrical parameters, such as fluid velocity, channel geometry, electrode shape and length, affect the current flowing at the electrode, the equilibration time, and the amount of analyte that is collected at the electrode [20,24,31,32,33,34,35,36,37,38,39]. A lot of effort has been made to model mass transport phenomena for microelectrodic systems, both for single-electrode configuration, or in presence of microelectrode arrays (possibly operating in different conditions, as in “digit” configuration) [24,40,41,42,43,44,45,46]. Theoretical models have been proposed along with numerical computations, mainly based on the finite elements analysis (FEA) [47,48,49]. More efficient modeling strategies (e.g., conformal mapping) and faster computing procedures based on GPU over CPU computing have also been examined [50,51].

These computational models face difficulties in their practical application, mainly because of the challenging process of providing them with sufficient versatility to match the situation of interest. Thus, a different formulation of the problem is required for any given geometry or physical and chemical properties of the system of interests [52].

In this paper, we showed how the non-dimensional formulation of the governing equations can be applied for the theoretical determination of the lower detection limits in microelectrodic systems. As the computational models are in non-dimensional form, we give results that can be adapted to different cases of study with the use of appropriate scaling factors. The set of data and trends derived from this work is thus proposed as a reusable tool for electrochemical modeling.

Stationary studies have been performed to consider the electrodic current in convective regime under variations of geometrical and physical parameters, and the trail effect generated by the presence of two consecutive electrode was evaluated [42,53,54]. Our simulations have been run using a commercial software implementing the finite element method, COMSOL^®^ Multiphysics.

## 2. Materials and Methods

### 2.1. Model Assumptions

A wide literature on the modeling strategy of electrochemical systems is available. Usually a system of partial differential equations involving Nernst-Planck and Navier-Stokes equations is set. In this context, our model considered an electrochemical reaction for which a fast charge transfer is assumed, so the electroactive species is completely depleted at the interface, and the current is only determined by transport. On this ground, with an appropriate scaling choice, the Nernst-Planck equation can be made dimensionless (Equation (1)):

(1) $${\tilde{c}}_{\tilde{t}}-\frac{1}{\mathrm{Pe}}{\tilde{\nabla }}^{2}\tilde{c}+\tilde{u}\cdot \tilde{\nabla }\tilde{c}=0$$

where Pe, $\tilde{u}$ and $\tilde{c}$ are respectively the Péclet number, the dimensionless velocity and concentration. Pe defines the relative importance of the diffusive and convective terms, i.e., when Pe approaches to 0 the diffusion dominates, while when Pe >> 1, convection becomes increasingly important. The velocity field that appears in Equation (1) is determined by the dimensionless Navier-Stokes equations (Equation (2)):

(2) {u˜t+(u˜⋅∇˜)u˜=−∇˜p˜+1ReΔ˜u˜∇˜∙u˜=0

where Re, $\tilde{u}$ and $\tilde{p}$ are respectively the Reynolds number, the dimensionless velocity, and pressure. It is worth mentioning that Re numbers for band microelectrode systems are generally much lower than 2000; hence, the flow inside the channel is laminar. For this reason, the flow profile at the channel inlet can be safely assumed to be parabolic.

### 2.2. Geometries and Discretization

**Figure 1:**
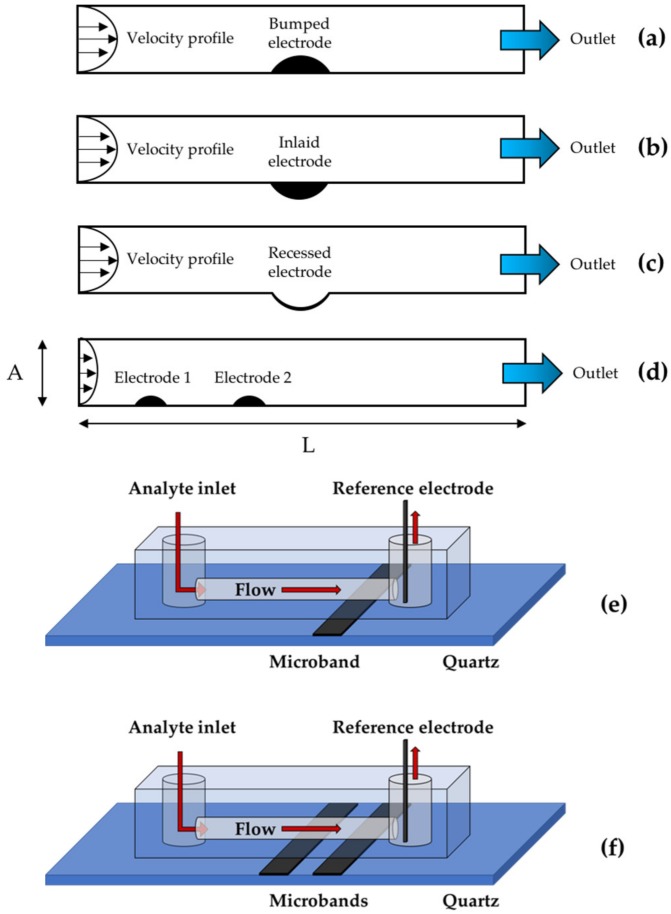
Cross sections of the channel geometries with one bumped (**a**), inlaid (**b**), or recessed (**c**) electrode and two bumped electrodes (**d**). Schemes of the devices containing single (**e**) and two-microbands electrode (**f**) (Working electrode not shown).

Each one of the three different types of microelectrodes systems is made of a channel where the electrodes lie at its bottom. In a typical geometry, electrodes are placed at the bottom of the channel (Figure 1). The domain is defined by 2D geometry where the third dimension z can be generated by translation when contour effects are negligible.

The channel length was set to L = 10 dimensionless units (du), and increased to L = 20 du when multielectrodic systems were simulated. The electrodic surface length was always constrained to 1 du. Channel height values have been swept from A = 1 to A = 5 du in unit steps for monoelectrodic stationary studies and when the trail effect was examined. The scheme of the flow and of the electroactive species in the microelectrodic channel considered in our studies can be summarized as in (Figure 1).

**Figure 2:**
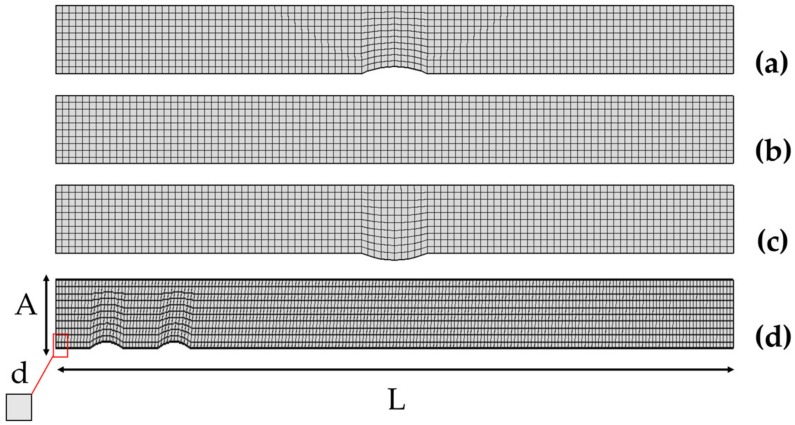
Geometry meshing for the bumped (**a**), inlaid (**b**), recessed (**c**), and two bumped electrodes (**d**) configurations. The channel length is represented by the L parameter, while the channel height is represented by the parameter A. The size of the square meshing elements is described in term of the mesh edge size d.

The Equations (1) and (2) for these geometries have been solved in the FEA framework where we applied a quadrangular discretization of the computational domain. The computational cells are regular squares with tunable edge size d (Figure 2) ranging from d = 0.1 to d = 0.01 du. In order to validate the discretization and to select the mesh density which ensures good accuracy and the lowest possible computation time, we have run different simulations with a completely developed laminar flow (Re = 1), and compared the electrodic dimensionless current values for different Pe numbers. Then, we selected the biggest d value that had a difference in dimensionless currents up to 1% when compared to the most accurate value d = 0.01 du, which was taken as reference (mesh convergence criterion).

## 3. Results

### 3.1. Validation of the Spatial Discretization

In this paragraph, the process applied for the choice of the meshing conditions that have been used throughout the modeling of the monoelectrodic and multielectrodic systems is reported.

**Figure 3:**
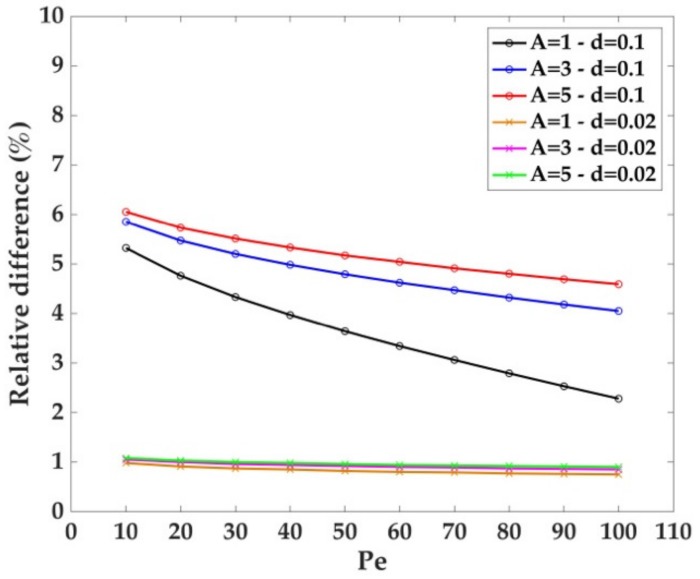
Mesh convergence criterion. Relative difference in electrodic dimensionless current compared with the most accurate d = 0.01 du taken as reference. Five channel heights were considered, namely A = 1, 2, 3, 4, and 5 du. The mesh size d = 0.02 du significantly reduced the discrepancy in current values with the reference mesh size d = 0.01 du, if compared with the less accurate mesh size d = 0.1 du.

According to the mesh convergence criterion described in the *Geometries and Discretization* session, the best value for the mesh size is d = 0.02 du (Figure 3). For the sake of clarity, Figure 3, reports the relative difference of dimensionless current with respect to the reference case where, d = 0.01 du. Higher d values brought an underestimation of the dimensionless currents. The relative difference of the dimensionless current (hereon relative difference) with respect to the reference case depends on the channel height (Figure 3). For instance, in the case of d = 0.1 du and Pe = 10, the relative difference changes from ~6% when A = 5 du to ~5% for A = 1 du (see Figure 3). When Pe = 100 the relative difference changes from ~5 to ~2%, considering the same channel heights. However, the relative difference between the currents at d = 0.02 du and d = 0.01 du is ~1% for each value of Pe, confirming the convergence of the mesh size. On this ground, d = 0.02 du was selected for all further simulations.

### 3.2. Single Electrode Case

Model systems were created starting from the simplest case of a single microelectrode embedded into a channel of dimensionless length L = 10 du, for which the stationary currents and collection efficiencies were derived. The data obtained were then analyzed with proper fitting functions to detect trends in these two key electrochemical parameters.

**Figure 4:**
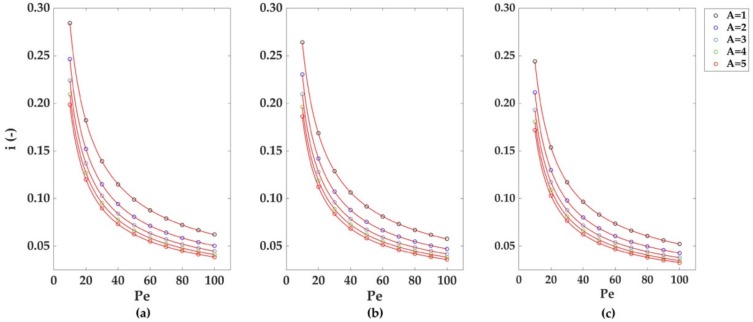
Dimensionless currents calculated for five different channel heights A = 1, 2, 3, 4, and 5 du for: (**a**) bumped (**b**) inlaid, and (**c**) recessed electrode geometries. For each of the three geometries considered, the dimensionless current values decreased as the Péclet number and the channel height increased. Mesh size d = 0.02 du. Channel length L = 10 du.

The simulations for the monoelectrodic case of study were run considering a perfect laminar flow (Re = 1), with an increasing relative importance of advective effects over diffusion effects (increasing Pe). Figure 4 depicts the dimensionless currents calculated for the three monoelectrodic geometries (a-b-c in Figure 2). The greatest dimensionless currents are obtained using a bumped geometry, and smaller values in channel height. The decrease of the dimensionless currents with Pe follows a power law decay; the result of the fitting is reported in Table 1a. The decrease of the dimensionless currents with the increase of channel height can also be described using a power law decay, with differences in currents coming closer to 0.02 du (dimensionless units) as Pe increases, as shown later. As reported in Table 2a, the recessed geometry exhibits the fastest reduction in current differences among the geometries, followed by the inlaid and recessed ones.

**Figure 5:**
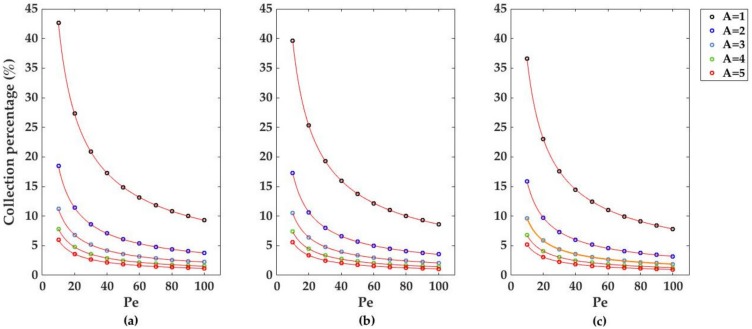
Collection percentage for five different channel heights, A = 1, 2, 3, 4, and 5 du, in the case of (**a**) bumped, (**b**) inlaid, and (**c**) recessed electrodes. Like the dimensionless electrodic currents in Figure 4, the collection percentages decreased as the Péclet number and the channel height were increased. Mesh size d = 0.02 du. Channel length L = 10 du.

**Figure 6:**
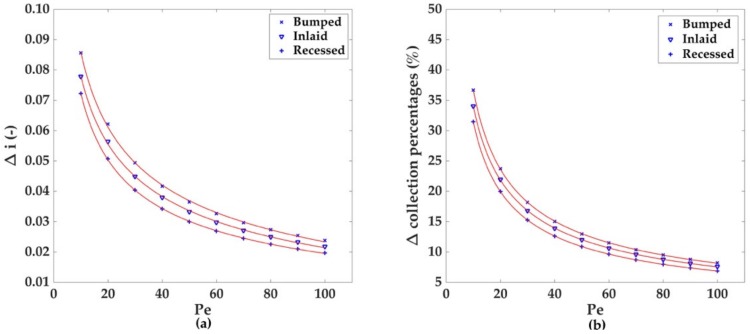
Difference between the values for the dimensionless currents (**a**) and collection percentages (**b**) obtained for A = 1 and A = 5 du at different values of the Péclet number. The data obtained for the three electrodic geometries were fitted using the power law decay $y=m∙x^{n}+c$ (Table 2).

Regarding the total percentage of species collected at the electrode for higher Pe values, three graphs are reported in Figure 5. For all the geometries, a strong decrease in the collection percentage occurs at increasing Pe and channel heights, with the bumped geometry being the most suitable for greater collection efficiencies. As with the case of the dimensionless currents, the decrease of the collection percentages with Pe follows a power law decay where the results of the fitting are listed in Table 1b. The decrease of the collection numbers with the increase of A and Pe can also be successfully described by the previous power law decay model used for the fitting of the dimensionless currents (Figure 6b, Table 2b); by comparison of the *n* values in the fitting model, a stronger dependence on Pe and A for the collection percentages and currents differences is highlighted.

### 3.3. Two Electrodes Case

**Figure 7:**
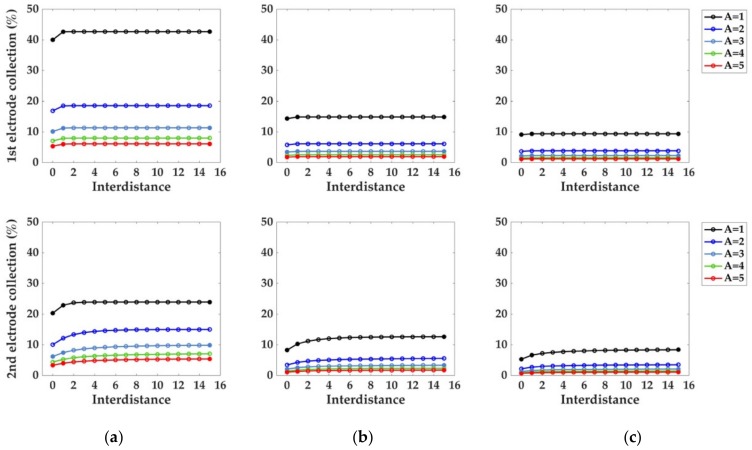
Collection percentages for Pe (**a**) 10, (**b**) 50 and (**c**) 100 calculated at the second electrode of the multielectrodic configuration. Five channel heights A were considered. The reported values were obtained setting the channel length to the fixed value of L = 20 du. Filled markers instead, represent control points from different simulations at A = 1, 3, 5 du which were performed to evaluate the presence of relevant border effects. This was achieved increasing the distances between the electrodes and the left and right borders of the channel, from 1 and 2 du to 4 and 9 du respectively (channel length L set to L = 30 du); no significant changes in collection percentages were detected. The mesh size d = 0.02 du was set according to the mesh convergence criterion of Figure 3.

After the previous characterization of monoelectrodic systems, the influence of multiple electrodes on the distribution of the electroactive species in the microchannel and on the collection percentages (trail effect) was investigated. Due to its higher current values and collection percentages, the bumped geometry was taken to better expose the trail effect, and a system consisting of two electrodes was selected for the simulations. The channel length was set as L = 20 du and the collection percentages at the first and second electrode for Pe = 10, 50 and 100 evaluated for channel heights A = 1, 2, 3, 4, and 5 du (Figure 7).

The electrodic interdistance, *f*, was increased from 0 to 15 du. To assess the absence of relevant truncation effects on the collection percentage, a set of greater distances between the electrodes and the left and right borders of the channel was also considered (filled markers in Figure 7).

**Figure 8:**
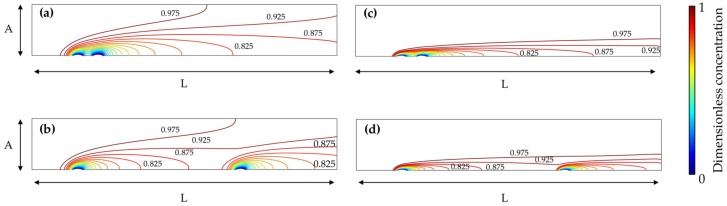
Contour plots of the dimensionless concentration for the bumped electrodic geometry with dimensionless channel height A = 5 du, dimensionless channel length L = 30 and variable electrode-electrode interdistance f for: (**a**) Pe = 10, f = 1; (**b**) Pe = 10, f = 15; (**c**) Pe = 100, f = 1; (**d**) Pe = 100, f = 15. Mesh size d = 0.02 du.

A graphical representation of the trail effect is given in Figure 8 for Pe = 10 and Pe = 100 where the computed distribution of the species in the domain of the two electrodes configuration shows that the convection boundary layers of the two electrodes interfere, as expected [31].

As illustrated from the graphs in Figure 7, for each Pe considered, the effect of the channel height on the collection percentage emerges as a dominant factor, with A = 1 du yielding the best performance, as lesser species are able to diffuse far from the electrodic surface.

Considering A = 1 du as particular example for the channel height, as the Péclet number is increased from 10 to 50 and 100, the electrodes need a greater interdistance for the collection number to become independent from the interdistance itself.

## 4. Discussion

The analysis of the mesh convergence lead to the observation of a clear trend where the smaller values of Pe and greater values of A and d contribute to the increase in computational error. The trends in Pe and A yield negligible effects when the mesh convergence criterion is matched.

Regarding the better performance of the bumped configuration in terms of higher electrodic currents, such behavior is known in literature [54]. Still, despite their lowest current values, we simulated the other two types of electrode, since they are widely available due to their easier production processes (e.g., recessed electrodes are easily produced with lithographic techniques [34]).

Concerning the two electrodes case, the lower collection number of the second electrode is usually strongly affected by the “trail effect”. This indicates that the superposition of the convection boundary layer causes the lower collection number of the second electrode. Comparing Figure 7 and Figure 8 we can, visually and quantitatively, confirm this causality relationship. Moreover, from the graphs in Figure 7, it is possible to see that after a certain interdistance (quantified in du), the superposition of the two concentration contour levels is reduced and the two electrodes can be considered as independent. From the graphs pertaining the second electrode, a counterintuitive trend emerges, as the increase in the interdistance necessary to reach this independence condition when Pe increases cannot be related to the size of the concentration contour levels, which, as shown in Figure 8, decreases when higher Pe numbers are considered. The first electrode, on the other hand, exhibits the expected trend of a faster independence condition as Pe is increased.

The importance of these results can be elucidated when noticing that the dimensionless formalism can be applied to any similar real system through a simple “pencil and paper” rescaling process. We want to stress that the use of such adaptation does not require the interested reader to perform any other simulation. Indeed, Equation (3) converts the dimensionless currents for the three monoelectrodic models (Figure 4) to predict the currents generated for a monoelectronic process (Equation (4)):

(3) $$i\;\left(A\right)=\tilde{i}∙\mathrm{Pe}∙D∙c^{0}∙z∙n∙F$$

(4) $$Q^{m+}+e^{-}⟶Q^{\left(m-1\right)+}$$

In Equation (3): $\tilde{i}$ represents the dimensionless electrodic currents; Pe is the Péclet number; *D* is the diffusion coefficient, which was set to *D* = 10^−9^ m^2^ s^−1^; *z* is the channel depth, which was set to 0.5 mm, *c*^0^ represents the bulk concentration which was taken as 0.1 mM (0.1 mol/m^3^), *n* represents the number of exchanged electrons, and *F* is the Faraday constant. From this same formula, an estimate of the lower detection limit can be extracted, (represented by *c*^0^)*,* provided the other physical and geometrical factors are known.

**Figure 9:**
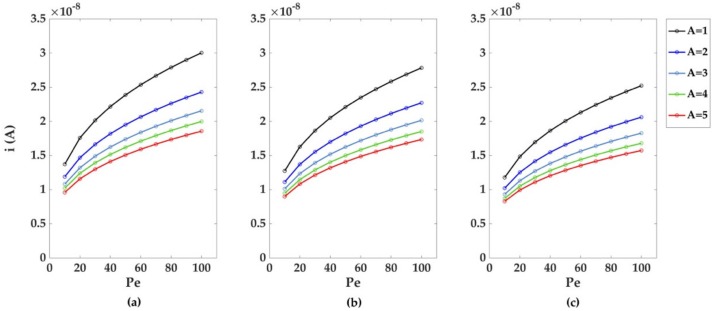
Dimensional currents for five different channel heights in the case of (**a**) bumped, (**b**) inlaid, and (**c**) recessed electrodes. The current values increased with increasing Péclet number value, and decreased as greater A values were considered.

Dimensionless and dimensional currents follow an opposite trend if greater Pe are considered, as the predominance of convective transport is translated in a reduced normal total flux at the electrodic surface, but also in a greater convective normal total flux at the electrodic surface (Figure 9).

The obtained dimensional currents lie in the range of tens of nanoamperes, and the highest values are obtained from bumped electrodic geometry, lower channel heights, and greater Péclet numbers.

Among all cases considered, differences in current values for varying channel heights did not significantly exceeded 1 × 10^−8^ A, even when greater values of Pe were considered. This confirm that a certain degree of freedom can be used while building a channel, without significant loss in terms of instrumental sensitivity.

**Figure 10:**
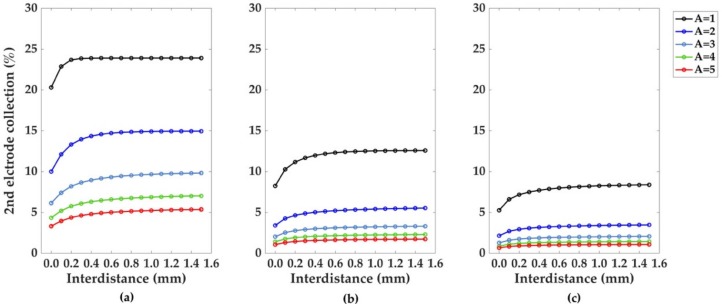
Trail effect. Dimensional model for the collection percentage at the second electrode, bumped configuration: (**a**) Pe = 10, (**b**) Pe = 50, (**c**) Pe = 100. Channel heights A = 1, 2, 3, 4, and 5, du were considered for the modeling and conversion to dimensional units. As for the monoelectrodic case, greater A values brought a reduction in the collection percentage of electroactive species at the second electrode.

Regarding the trail effect electrodic interdistance, the conversion in dimensional units is obtained by Equation (5):

(5) $$x=\;L\prime ∙\tilde{x}$$

where *L’* is the scaling factor used in the dimensionless formulation of the convective diffusive problem. If *L*’ is set to 100 μm, and considering the collection percentages at the second electrode, the interdistances reported in Figure 7 can be converted as in Figure 10, with distance values that are encountered in real cases of study [30]. If without loss of generalization the A = 1 du parameter from Figure 10 is considered, the interdistance required for the electrode to become independent of each other, and their respective plateau in collection percentage can be derived, as in Table 3, showing how a greater interdistance between consecutive electrodes is required to reach the independence condition. This trend deriving from the presence of consecutive electrodes is counterintuitive if described only by concentration gradients, and it will be studied within the dimensionless formalism in successive analysis.

## 5. Conclusions

In this work, we have modeled the mass charge transport on channel with band microelectrodes by means of the Finite Element Analysis, as implemented on the commercial software package COMSOL^®^ Multiphysics. Taking the simplest possible case of study (the monoelectrodic one), we have analyzed stationary non-dimensional computational models whose result are not limited to a particular set of parameters. The quantitative validation of the spatial discretization we applied to the governing equations, has also been offered.

An example has been reported showing how the values for the electrodic current can be computed from these results. Next, the obtained current values may be used to estimate the current range expected for microelectrodic detectors when a definite amount of electroactive species is considered. The lower detection limit of the instrument can thus be considered from the experimenter while choosing the proper electrode for measurements.

The bumped geometry, with the electrode protruding from the channel surface is known for its better collection percentage and higher current yield. This characteristic has been observed from the dimensionless point of formulation of the diffusive-convective problem [54]. A successive step has been that of confirming the “trail effect” arising from the positioning of successive microelectrodes in the channel. This is considered a relevant factor for the optimization of the microelectrodes devices [31]. We determined how geometric parameters as interelectrodic distance and channel height can affect the collection percentage of the electroactive species. For the general case of two consecutive electrode, we have observed how a larger interdistance is required for the second electrode to become independent from the first one as greater Pe number are considered; this is an apparently counterintuitive trend if concentration contour levels only are considered.

Finally, we showed how our results can be rescaled to any equivalent problem without the need to run new computation, while giving practical information (dimensional currents and detection limits). It is the opinion of the authors that this can contribute to the process of microelectrodic device construction, where our data can be exploited as technical references.

## Figures and Tables

**Table 1:** Fitting of the dimensionless currents (**a**) and collection percentages (**b**) reported in Figure 4 and Figure 5 respectively. For each of the three considered geometries, the values have been fitted using the function $y=m∙x^{n}+c$. R represents the fitting error.

(a)					(b)				
	m	c	n	R		m	c	n	R
Convex					Convex				
A = 1	1.2416	−0.0060	−0.6310	1.0000	A = 1	186.2379	−0.8936	−0.6310	1.0000
A = 2	1.2310	0.0018	−0.7019	1.0000	A = 2	92.3260	0.1353	−0.7019	1.0000
A = 3	1.1625	0.0025	−0.7201	1.0000	A = 3	58.1252	0.1262	−0.7201	1.0000
A = 4	1.1124	0.0028	−0.7313	1.0000	A = 4	41.7144	0.1037	−0.7313	1.0000
A = 5	1.0745	0.0029	−0.7395	1.0000	A = 5	32.2325	0.0860	−0.7395	1.0000
**Flat**					**Flat**				
A = 1	1.1677	−0.0042	−0.6386	1.0000	A = 1	175.1537	−0.6334	−0.6386	1.0000
A = 2	1.1550	0.0020	−0.7043	1.0000	A = 2	86.6302	0.1516	−0.7043	1.0000
A = 3	1.0942	0.0027	−0.7231	1.0000	A = 3	54.7112	0.1327	−0.7231	1.0000
A = 4	1.0494	0.0029	−0.7349	1.0000	A = 4	39.3508	0.1072	−0.7349	1.0000
A = 5	1.0153	0.0029	−0.7436	1.0000	A = 5	30.4582	0.0881	−0.7436	1.0000
**Concave**					**Concave**				
A = 1	1.1349	0.0005	−0.6666	1.0000	A = 1	170.2478	−0.0669	−0.6666	1.0000
A = 2	1.0792	0.0022	−0.7123	1.0000	A = 2	80.4925	0.1623	−0.7123	1.0000
A = 3	1.0259	0.0026	−0.7317	1.0000	A = 3	51.2951	0.1323	−0.7317	1.0000
A = 4	0.9864	0.0028	−0.7439	1.0000	A = 4	36.9915	0.1044	−0.7439	1.0000
A = 5	0.9649	0.0033	−0.7576	1.0000	A = 5	28.6915	0.0847	−0.7576	1.0003

**Table 2:** Fitting parameters, $y=m∙x^{n}+c$ used for the (**a**) dimensionless currents and (**b**) collection percentages differences of Figure 6. R represents the fitting error.

(a)					(b)				
	m	c	n	R		m	c	n	R
convex	0.2564	−0.0226	−0.3734	0.9994	convex	155.0329	−1.0664	−0.6133	1.0000
flat	0.2334	−0.0192	−0.3800	0.9995	flat	145.6101	−0.7958	−0.6212	1.0000
concave	0.2313	−0.0094	−0.4518	1.0000	concave	142.1452	−0.1940	−0.6523	1.0000

**Table 3:** Trail effect. Dimensional parameters obtained from the second electrode collection percentages reported in Figure 10. Reference channel height, A = 1 du (scaled to A = 100 µm in dimensional form).

Pe	Interdistance (mm)	Collection (%)
10	0.3	24
50	0.8	13
100	1.3	8
